# Bacteriophage and Endolysin Encapsulation Systems: A Promising Strategy to Improve Therapeutic Outcomes

**DOI:** 10.3389/fphar.2021.675440

**Published:** 2021-05-07

**Authors:** Vijay Singh Gondil, Sanjay Chhibber

**Affiliations:** ^1^ Department of Microbiology, Basic Medical Sciences, Panjab University, Chandigarh, India; ^2^ Department of Nephrology, Postgraduate Institute of Medical Education and Research, Chandigarh, India

**Keywords:** bacteriophages, endolysins, encapsulation, nanoparticles, antibiotic resistance, alternative therapy

## Introduction

Bacteriophages (phages) are bacterial predators which shape bacterial population dynamics in nature by rapidly killing their host bacterium (Abedon, 2008; Clokie et al., 2011). Phages were discovered at the beginning of the 20th century and explored for their therapeutic potential to treat human and animal infections (Duckworth and Gulig, 2002). However, the discovery of antibiotics and their rapid development in subsequent years declined the interest in the therapeutic application of phages (Sulakvelidze and Morris, 2001). In recent decades, the emergence of multidrug-resistant bacterial pathogens has spurred clinicians and researchers to look for alternative therapeutic options, which mainly includes phages. Phages have been evaluated in various animal models and clinical trials to establish their clinical relevance in treating drug-resistant bacterial infections (Abdelkader et al., 2019; Brix et al., 2020; Pirnay and Kutter, 2020). Based on the results of previous studies, these entities are considered as fascinating future antimicrobial agents. Interestingly, several pharmaceutical companies are actively engaged in commercializing phage-based therapeutics (Report, 2021). Along with bacteriophages, phage borne lytic proteins known as endolysins have also been extensively investigated in the past few years. Endolysins possess numerous advantageous properties over whole phage entities, which primarily are high specificity, rapid host lysis, modular structure, and low chances of resistance (Gondil et al., 2020b). Endolysins have also been evaluated in several animal infection models as well as phase I and phase II clinical trials to establish their efficacy for the treatment of drug-resistant bacterial infections (Abdelkader et al., 2019; Gondil et al., 2020b). Despite the therapeutic effects of phages and endolysins, these alternative agents face some empirical hurdles posed by the host system, which include low bioavailability, loss of activity, non-targeted delivery, rapid clearance by the reticuloendothelial system and antibody-mediated inactivation (Loh et al., 2020). Authors have also experienced and reported similar limitations in phage and endolysin mediated treatment of animal infection models in their studies (Singla et al., 2015; Singla et al., 2016; Gondil et al., 2021). Against this backdrop, a resurgent interest has been seen among researchers to evaluate the potential of delivery systems for encapsulation of bacteriophages and endolysins. A plethora of phage and endolysin encapsulation techniques have been reported in recent years (Loh et al., 2020). These delivery systems are being exploited for the treatment of acute and chronic infections in animal models by improving pharmacokinetic parameters and altered host immune response against therapeutic entities (Gondil and Chhibber, 2017).

## Phage and Endolysin Encapsulation Systems

Several phage encapsulation studies have explored the potential of various drug delivery systems, which primarily include natural polymers, synthetic polymers, liposomes and electrospun fibers. Polymeric phage encapsulation has been extensively studied in the treatment of gastrointestinal tract infections. These polymers protect phages from harsh acidic conditions, potentially leading to phage inactivation or loss of phage titer. Other than extreme conditions, these polymeric encapsulation materials also protect encapsulated phages from digestive enzymes, bile juices and provide permeability to mucous lining where bacterial pathogens may reside (Malik et al., 2017). Numerous intrinsic properties of natural polymers such as limited sensitivity to enzymatic degradation, pH responsiveness, capable of crosslinking and tailor-made designing also render them a suitable candidate for phage delivery systems. In the gastrointestinal tract, natural polymeric materials such as chitosan, alginate, whey protein provide a safeguard to phages by improving their survival, which in turn increases their therapeutic efficacy (Ma et al., 2008; Dini et al., 2012; Tang et al., 2013; Colom et al., 2017). In pursuit of targeted delivery, Vinner and Malik (2018) demonstrated the alginate containing pH-responsive microencapsulation system triggered the controlled release of active Felix O1 phages in the gastrointestinal tract. Some natural cationic polymers, which primarily include chitosan, are also recognized to possess antiviral properties and reduces the bacteriophage concentration up to 1–2 logs in 1 min of phage-polymer coincubation (Ly-Chatain et al., 2013). These polymeric materials may interfere with phage receptors and modulate electrostatic interaction to impair their adsorption on their host cells, thus decreasing the infectivity and titer of phages. The polymer-phage interaction should be pre-evaluated in different physicochemical parameters to tackle the polymer mediated inactivation of bacteriophages.

Similarly, synthetic polymers such as poly (lactic-co-glycolic acid and methacrylate-comethacrylic acid-based phage encapsulation delivery systems were also developed to augment gastrointestinal delivery (Puapermpoonsiri et al., 2009; Stanford et al., 2010). Encapsulated phage preparations exhibited high resistance to gastric acid and reduced fecal shedding of pathogenic bacteria in infection-induced animals. In the last few years, synthetic polymers are also being used for designing smart phage encapsulation systems in the treatment of topical infections, which can release the therapeutic payload on the specific physiochemical trigger. Such systems can be designed using thermo-responsive materials as *Staphylococcus aureus* phage K was encapsulated in poly (N-isopropylacrylamide)- allylamine nanospheres. These nanospheres undergo a temperature-dependent phase transition to release phage at an elevated temperature associated with bacterial infections (Hathaway et al., 2015). Another smart system class is “pH-sensitive formulations,” which allows the release of loaded phages at a specific pH. A pH-responsive polymer polymethyl methacrylate-co-methacrylic acid was employed to release *Proteus mirabilis* phage from urinary catheters in response to elevated urinary pH associated with bacterial infection (Milo et al., 2016). A similar system that uses the hyaluronidase enzyme as a trigger to release phages has also been reported in the literature. *S. aureus* phages were encapsulated in a layer of agarose and hyaluronic acid methacrylate (HAMA) polymer, which is sensitive to hyaluronidase. During infection state, HAMA is solubilized by hyaluronidase (produced from *S. aureus*) and release phages in the vicinity of pathogens to clear the infection (Bean et al., 2014). Synthetic polymers exert high control in the delivery of therapeutic entities in a range of physicochemical conditions. As compared to natural polymers, encapsulation in synthetic polymers involves the usage of organic solvents, which may decrease the phage concentration or infectivity over time. Phage and solvent choice must be compatible and needs to be pre-evaluated to ensure the therapeutic efficacy of encapsulated phages. In the treatment of gastrointestinal, respiratory, and intracellular pathogens, lipid-based encapsulation of phages in the form of liposomes has been studied extensively in the literature. In oral delivery, other than protection from gastric conditions, liposomes promote mucoadhesiveness for increased retention time in the intestine, which increases the efficacy of phage preparation (Colom et al., 2015). In our laboratory, Singla et al. established the efficacy of encapsulated phage liposome preparation in treating *K. pneumoniae* induced pneumonia in the murine model. Superior therapeutic efficacy and increased phage concentration was seen in the blood and other organs over a period of time. This observation supports the altered pharmacokinetic behavior and immunological response toward encapsulated over nonencapsulated phages (Singla et al., 2015; Singla et al., 2016). Liposomal phage preparation also showed 100% unresponsive to neutralizing antibodies and retained lytic activity into macrophage by intracellular localization, which is uncommon for native free phages (Singla et al., 2016). In other studies from our laboratory, liposomes and their modified counterparts transferosomes were also evaluated for successful treatment of burn wound and soft tissue infections in animal models (Chadha et al., 2017; Chhibber et al., 2017). From the clinical point of view, the intracellular delivery of phages is critical in treating drug-resistant intracellular pathogens as free phages have limited ability to move across the plasma membrane. Lipid-based encapsulation provides a “Trojan horse strategy,” which involves lipid carrier-mediated intracellular delivery of phages into the host cell to eradicate intracellular pathogens. The lipid-based phage cargos can be further explored for better therapeutic outcomes, including the use of other lipoidal delivery systems (ethosomes, niosomes, and transferosomes), targeted delivery, stimuli-responsive delivery, blending of hydrophilic polymers for increased retention, and positively charged polymers to aid mucosal adhesion. Organic solvents such as chlorinated solvents, ethanol, ethyl acetate, diethyl ether and methanol are generally used in the synthesis of liposomes. These solvents are removed from the formulations by evaporation; however, some traces of these solvents in the final preparation may lead to loss of phage titer, infectivity and presents a risk to human health. The use of advanced evaporation techniques, which includes reverse-phase evaporation, tangential flow filtration and rotatory evaporation methods, efficiently removes the solvent without influencing the stability of liposome formulations. Moreover, phage compatible and less toxic organic solvents such as todiethyl ether can be employed to maintain the therapeutic efficacy and limit the toxicity associated with the phage liposomal formulations. Electrospun phage delivery means encapsulation of phages in polymeric fibers (cellulose diacetate, polyethylene oxide, polyvinyl pyrrolidone and polyvinyl alcohol) using electrospinning (Malik et al., 2017). Electrospun phage preparations are being investigated in the *in-vitro* models and have shown retained activity of the encapsulated phages. Polycaprolactone electrospun nanofibers based phage encapsulation also showed a 99.99% decrement of *Pseudomonas aeruginosa* population in 2 h (Nogueira et al., 2017). In a recent study, Phagestaph and Fersis phages were encapsulated and evaluated for their biocompatibility and antibacterial activity against *S. aureus* and *Streptococcus pyogenes* (Díaz et al., 2018). However, the electrospun phage delivery system’s therapeutic applicability is limited to *in-vitro* experiments requiring more extensive validation, especially in animal infection models. In our experience, phage delivery systems are considered extremely effective in the early stages of infection, where only a single dose of phage formulation can significantly eradicate the infection. However, late administration of phage formulation requires co-administration of antibiotic or multiple doses of phage formulation to combat the infection progression.

Endolysins, a class of bacteriolytic phage borne proteins, have also been encapsulated to enhance their therapeutic potential. However, unlike phage encapsulation, endolysin delivery systems are still in a very juvenile stage. Endolysin delivery strategies are considered more challenging than phage delivery because of the proteinaceous nature of endolysins and labile enzymatic activity. Organic solvents and harsh encapsulation conditions may affect the structure and function of these enzymatic entities. To date, limited studies have reported the delivery of endolysins in a truncated or full-length version for their controlled delivery and higher antibacterial activity. Hathaway et al. reported the truncated cysteine histidine-dependent amino hydrolase/peptidase (CHAP_K_) domain of LysK endolysin and lysostaphin in a thermally triggered Poly (N-isopropylacrylamide) (PNIPAM) nanoparticles for delivery of CHAP_K_ cargo at a higher temperature, which is a standard indicator of infection (Hathaway et al., 2015). These delivery carriers can be designed for the delivery of antimicrobial cargos at a specific temperature which may be associated with a particular bacterial infection. Along with the development of phage delivery systems, our laboratory has extensively explored the delivery systems for anti-streptococcal and anti-staphylococcal endolysins. An anti-streptococcal full-length Cpl-1 endolysin was loaded into mucoadhesive chitosan nanoparticles for their pulmonary delivery and high antibacterial activity (Gondil et al., 2020a). Cpl-1 loaded into chitosan nanoparticles showed higher antibacterial efficacy than Cpl-1 alone in an *in-vitro* as well as animal infection model (Gondil et al., 2020a; Gondil et al., 2021). Encapsulation of Cpl-1 in chitosan nanoparticles enhanced its bioavailability and provided substantial mucoadhesiveness, which could be one of the major contributing factors in eliminating pulmonary bacterial infection. Portilla et al. reported the encapsulation of LysRODI endolysin in pH-sensitive liposomes, which demonstrate the ability of liposomes for targeted delivery of endolysin under mild acidic conditions. Encapsulated LysRODI was shown to significantly effective in reducing the cell count of *S. aureus* (planktonic and biofilm form) at pH 5 (Portilla et al., 2020). Recently, Kaur et al. also reported a chitosan-alginate based endolysin delivery system for efficient delivery of LysMR-5, an anti-staphylococcal endolysin that still needs to be evaluated in animal infection models (Kaur et al., 2020). In general, chitosan-based formulations are favorable choice over other type of delivery materials to deliver biotherapeutics. Chitosan-based delivery systems not only enhance the bioavailability of therapeutic agents but also clear safely from the host system after delivery of their loaded cargos.

Despite the high therapeutic potential, phages and endolysin delivery systems face multiple challenges in their clinical implications. Ideal phage or endolysin concentration, their pharmacokinetics and immunomodulatory properties are critical knowledge gaps in the understanding and applications of phage and endolysin delivery systems. The comprehensive architectural range of phages (tailed and non-tailed) and endolysins (single modular and multi-modular) may lead to considerable variation in the formulation design, release kinetics and therapeutic outcome. Thus, delivery systems are needed to be critically designed and optimized for each phage or endolysin, which may increase the scaling-up efforts and cost in developing such formulations. Sterilization of prepared formulations is a major challenge in the scale-up of these delivery systems as thermosensitive phages, and endolysins cannot undergo high-temperature sterilization. The use of sterile conditions for deigning formulations or UV treatment is another potential alternative, but it also increases the cost as well as logistics difficulties in large-scale production settings. The natural origin of phages categorizes phages into non-patentable entities, making them less profitable and unappealing for pharmaceutical companies. Apart from technological limitations, a regulatory and legal framework is another major hurdle in the clinical progress of phage and endolysin delivery systems. The regulatory and legal framework for phages and endolysin applications is more limited to the local, nationwide boundaries and is yet to be standardized in a global manner (Furfaro et al., 2018). Phage and endolysins delivery systems are also expected to enter the clinical trials in the near future to establish their clinical acceptance. It is vital that scientists, clinicians, and regulatory bodies must work together to make the appearance of these therapeutic preparations in clinics sooner rather than later.

## Future Perspectives

The future of phage and endolysin encapsulation systems seems to be bright. With the emergence of antimicrobial resistance, the phage and their antibacterial products have emerged from a forgotten area to become a central research area for healthcare workers. There is a need to make these products more effective as therapeutic agent for treating human infections. Various delivery strategies have been devised to increase the efficacy and applicability of these antimicrobial products to clinical care. Phages have been evaluated in multiple encapsulation systems; however, there is a long road for the endolysins delivery system to prove their therapeutic applicability. Phage and endolysin delivery systems must undergo through a range of animal validation, followed by well-structured large clinical trials to establish their therapeutic outcome. The most obvious challenge for clinical applications of phage and endolysins delivery is the lack of a uniform regulatory framework across the globe. Progression of clinical trials and more scientific evidence may refine the current regulatory processes to a well-structured regulatory and legal framework. These future initiatives can further improve the understanding as well as clinical acceptance of phage and endolysin delivery systems to alleviate the burden of bacterial infections in healthcare. The current antibiotic crisis has driven the shifting of paradigm to phage-based therapies and countering delivery challenges. The next 1–2 decades could be an intriguing time to develop novel phage and endolysin delivery systems and investigate their potential in the healthcare system to combat multidrug-resistant infections.
